# Effects of antibiotics on the *in vitro* expression of tetracycline-off constructs and the performance of *Drosophila suzukii* female-killing strains

**DOI:** 10.3389/fbioe.2023.876492

**Published:** 2023-02-14

**Authors:** Ying Yan, Bashir Hosseini, Annemarie Scheld, Srilakshmi Pasham, Tanja Rehling, Marc F. Schetelig

**Affiliations:** ^1^ Department of Insect Biotechnology in Plant Protection, Institute for Insect Biotechnology, Justus-Liebig-University Giessen, Giessen, Germany; ^2^ Liebig Centre for Agroecology and Climate Impact Research, Justus-Liebig-University Giessen, Giessen, Germany

**Keywords:** genetic control, transgenic sexing strain, tetracycline-off system, *Drosophila suzukii*, sterile insect technique, 2A peptide

## Abstract

Genetic control strategies such as the Release of Insects Carrying a Dominant Lethal (RIDL) gene and Transgenic Embryonic Sexing System (TESS) have been demonstrated in the laboratory and/or deployed in the field. These strategies are based on tetracycline-off (Tet-off) systems which are regulated by antibiotics such as Tet and doxycycline (Dox). Here, we generated several Tet-off constructs carrying a reporter gene cassette mediated by a 2A peptide. Different concentrations (0.1, 10, 100, 500, and 1,000 μg/mL) and types (Tet or Dox) of antibiotics were used to evaluate their effects on the expression of the Tet-off constructs in the *Drosophila* S2 cells. One or both of the two concentrations, 100 and 250 μg/mL, of Tet or Dox were used to check the influence on the performances of a *Drosophila suzukii* wild-type strain and female-killing (FK) strains employing TESS. Specifically, the Tet-off construct for these FK strains contains a *Drosophila suzukii nullo* promoter to regulate the *tetracycline transactivator* gene and a sex-specifically spliced pro-apoptotic gene *hid* ^*Ala4*^ to eliminate females. The results suggested that the *in vitro* expression of the Tet-off constructs was controlled by antibiotics in a dose-dependent manner. ELISA experiments were carried out identifying Tet at 34.8 ng/g in adult females that fed on food supplemented with Tet at 100 μg/mL. However, such method did not detect Tet in the eggs produced by antibiotic-treated flies. Additionally, feeding Tet to the parents showed negative impact on the fly development but not the survival in the next generation. Importantly, we demonstrated that under certain antibiotic treatments females could survive in the FK strains with different transgene activities. For the strain V229_M4f1 which showed moderate transgene activity, feeding Dox to fathers or mothers suppressed the female lethality in the next generation and feeding Tet or Dox to mothers generated long-lived female survivors. For the strain V229_M8f2 which showed weak transgene activity, feeding Tet to mothers delayed the female lethality for one generation. Therefore, for genetic control strategies employing the Tet-off system, the parental and transgenerational effects of antibiotics on the engineered lethality and insect fitness must be carefully evaluated for a safe and efficient control program.

## Introduction

Genetic control strategies that introduce controllable sterility or lethality into the insect population have been developed in several insect species, including agricultural pests and human disease vectors (Harvey-Samuel et al., 2017; Alphey and Bonsall, 2018). Those strategies often mimic the principle of the sterile insect technique (SIT), which releases a large number of radiation-sterilized insects to suppress or eradicate the targeted pest population (Knipling, 1955; Klassen and Vreysen, 2021). Unlike classical SIT approaches that use wild-type strains and genetic sexing strains (GSSs) generated by classical genetics for mass-production, strategies such as Release of Insects Carrying a Dominant Lethal (RIDL) gene and Transgenic Embryonic Sexing System (TESS) took advantages of modern genetic engineering techniques. They generated insect strains carrying fluorescent gene marker(s) that could facilitate the monitoring of a release program and used lethal genes that kill insects at certain stages (Häcker et al., 2021). In addition, the tetracycline-off (Tet-off) system was used in those RIDL and TESS strains to control the expression of the lethal genes and allow population maintenance.

The conditionality of a Tet-off system is based on the interaction between the tetracycline transactivator (tTA) protein and the tetracycline response element (TRE), which can be intercepted by Tet or its derivatives such as doxycycline (Dox) (Gossen and Bujard, 1992; Berens and Hillen, 2003). When Tet is not present, tTA activates the expression of the TRE-linked effector gene, which was successfully demonstrated in *Drosophila melanogaster* (Bello et al., 1998). To introduce the controllable lethality, a cytotoxic or pro-apoptotic gene was linked to TRE to generate a Tet-repressible RIDL system in *Drosophila melanogaster* (Heinrich and Scott, 2000; Thomas et al., 2000). It was further discovered that *tTA* itself could be used as an effector gene to build an autoregulation loop, leading to the accumulation of tTA (Gong et al., 2005). Such overexpression of tTA typically killed insects at late developmental stages, such as late larvae or pupae, although the actual lethal mechanism behind it is still unknown (Phuc et al., 2007; Ant et al., 2012; Li et al., 2014; Concha et al., 2016). For TESS, promoters from cellularization genes were used to drive tTA, which activate the female-specific (fs) pro-apoptotic gene expression and kill all female insects at embryonic or early larval stages due to the induced apoptosis. The TESS strains were generated in tephritid fruit flies such as *Ceratitis capitata*, *Anastrepha suspensa*, and *A. luden*s (Schetelig and Handler, 2012b; Ogaugwu et al., 2013; Schetelig et al., 2016), as well as calliphorid blowflies such as *Lucilia cuprina* and *Cochliomyia hominivorax* (Yan and Scott, 2015; Yan et al., 2020b; Concha et al., 2020), which could reduce the rearing cost of a control program. Like TESS, female-specific RIDL can also be generated by using an fs-promoter for *tTA* or an fs-spliced intron for the effector gene (Thomas et al., 2000; Fu et al., 2007; Jin et al., 2013). Those female-killing (FK) strategies could lead to male-only releases, which are more effective than bi-sexual releases for an SIT program (Rendon et al., 2004; Schliekelman et al., 2005; Franz et al., 2021).

For an FK strategy using a Tet-off system, the regulator Tet plays a decisive role in the sexing efficiency and strain fitness. For example, the engineered insect lethality was directly linked to the concentrations of the Tet derivatives (Thomas et al., 2000; Horn and Wimmer, 2003). The lethal stages were also dependent on the timing of Tet removal (Gong et al., 2005; Schetelig et al., 2009; Yan and Scott, 2015), and the lethal phenotype can be reversed by adding Tet again (Horn and Wimmer, 2003; Schetelig et al., 2016). While low concentrations of Tet were not enough to suppress binding between tTA and TRE and the resulting lethality, high concentrations of Tet showed a deleterious effect on insect fitness (Schetelig et al., 2009; Schetelig and Handler, 2012a; Yan and Scott, 2015; Concha et al., 2020). We previously generated transgenic FK strains employing a Tet-off system in the spotted wing *Drosophila* (*Drosophila suzukii*; Diptera, Drosophilidae), a devastating fruit fly that recently became highly invasive (Asplen et al., 2015; Dos Santos et al., 2017). The Tet-off construct V229 that used to generate these FK strains contains an embryonic promoter from *Drosophila suzukii nullo* gene to regulate the *tTA* (driver cassette) and a sex-specifically spliced pro-apoptotic gene *hid* ^*Ala4*^ (effector cassette) to eliminate female flies. While the most efficient FK strain eliminated all-female offspring at the embryonic stage if Tet was only fed to the larvae in the parental generation, some other strains killed the majority of females at late stages (Schetelig et al., 2021). It was speculated that Tet could be maternally inherited and switch off the embryonic lethality (Horn and Wimmer, 2003; Schetelig et al., 2009; Schetelig and Handler, 2012a; Yan et al., 2017). Here, we generated a series of Tet-off constructs that contain the same drive cassette as in V229 and a reporter gene cassette in which EGFP-NLS and DsRed-CAAX are co-expressed by a 2A peptide. By testing the Tet-off reporter constructs *in vitro* and *D. suzukii* WT or FK strains *in vivo*, we aim to investigate 1) how the antibiotics regulate the expression of Tet-off constructs at the cell level, 2) how the antibiotics affect the fly development and survival in the next generation, and 3) whether the antibiotics could suppress or delay the engineered lethality in our FK strains with different transgene activities.

## Methods and materials

### Insect rearing

The *Drosophila suzukii* wild-type (WT) USA strain and transgenic lines were maintained at 25°C and 55%–60% humidity under a 12L/12D photoperiod (Schwirz et al., 2020; Schetelig et al., 2021). The WT-USA strain was reared on an antibiotic-free diet, and the transgenic strains were kept on the same diet supplemented with 100 μg/mL Tet (Thermo Fisher Scientific).

### Plasmid construction

Bicistronic gene cassettes containing EGFP-NLS (or DsRed-NLS) and DsRed-CAAX (or EGFP-CAAX), which were separated by a 2A peptide (DrosCV-2A or TaV-2A), were excised from V220*_pBXLII_attP_PUb-AmCyan_PUb-DsRed-NLS-DrosCV-2A-EGFP-CAAX-SV40*,V221*_pBXLII_attP_PUb-AmCyan_PUb-DsRed-NLS-TaV-2A-EGFP- CAAX-SV40*,V222*_pBXLII_attP_PUb-AmCyan_PUb-EGFP-NLS-DrosCV-2A-DsRed-CAAX-SV40*, or V223*_pBXLII_attP_PUb-AmCyan_PUb-EGFP-NLS-TaV-2A-DsRed-CAAX-SV40* (Schwirz et al., 2020) and used to replace the DsRed-NLS in the vector V206*_pXLBacII-attP-PUb-AmCyan_Dsnullo_DsRed-NLS-SV40* (Yan et al., 2020a) at the Bsu36I and MluI restriction sites, to generate V355*_pBXLII_attP_PUb-AmCyan_Dsnullo-DsRed-NLS-DrosCV-2A-EGFP-CAAX-SV40*,V356*_pBXLII_attP_PUb-AmCyan_Dsnullo-DsRed-NLS- TaV-2A-EGFP-CAAX-SV40*, V357*_pBXLII_attP_PUb-AmCyan_Dsnullo-EGFP-NLS-DrosCV-2A-DsRed-CAAX-SV40*, and V358*_pBXLII_attP_PUb-AmCyan_Dsnullo-EGFP-NLS-TaV-2A-DsRed-CAAX-SV40*, respectively. Similarly, the excised bicistronic gene cassette from V220, V221, V222, or V223 was used to replace the effector gene cassette *Dshid* ^*Ala4*^ *-CctraF* in the vector V229*_pBXLII_attP_PUbAmCyan_Dsnullo-tTA-SV40_TREhs43-Dshid* ^*Ala4*^ *-CctraF-SV40* (Schetelig et al., 2021) at the Bsu36I and MluI restriction sites to generate V359*_pBXLII_attP_PUbAmCyan_Dsnullo-tTA-SV40_TREhs43-DsRed-NLS-DrosCV-2A-EGFP-CAAX-SV40*, V360*_pBXLII_attP_PUbAmCyan_Dsnullo-tTA-SV40_TREhs43-DsRed-NLS-TaV-2A-EGFP-CAAX-SV40*, V361*_pBXLII_attP_PUbAmCyan_Dsnullo-tTA-SV40_TREhs43-EGFP-NLS-DrosCV-2A-DsRed-CAAX-SV40*, and V362*_pBXLII_attP_PUbAmCyan_Dsnullo-tTA-SV40_TREhs43-EGFP-NLS-TaV-2A-DsRed-CAAX-SV40*, respectively.

### Cell culture experiments

The cell transfection and counting were carried out as previously described (Yan et al., 2020a; Schetelig et al., 2021). Briefly, *Drosophila* Schneider 2 (S2) cells were grown on Schneider’s medium containing 10% heat-inactivated fetal bovine serum (Hi-FBS) and 1% penicillin/streptomycin in closed-capped flasks without CO_2_ at 25°C. The Xfectin Transfection Reagent (Takara), 1 μg of plasmid DNA, and Tet or Dox (Alfa Aesar) at 0.1, 10, 100, 500, or 1,000 μg/mL (correspondingly named Tet-0.1, Tet-10, Tet-100, Tet-500, Tet-1000, Dox-0.1, Dox-10, Dox-100, Dox-500, and Dox-1000) were used for transient transfections. A 13-mm TC coverslip (Sarstedt) was placed into each well of a 24-well plate to facilitate imaging. The transfection was stopped by refreshing the dishes with 500 µL of Schneider’s medium containing 10% Hi-FBS and 1% penicillin/streptomycin. The cells were incubated for ∼18 h at 25° C before fixing in 4% paraformaldehyde for 15 min and washing briefly with PBS prior to microscopy. The transfected cells were imaged using a M205FA microscope (Leica Microsystems) with filter sets CFP for AmCyan (ex. 436/20; em. 480/40), YFP for EGFP (ex. 510/20; em. 560/40), TxRed for DsRed (ex. 545/30; em. 620/60), and GFP-LP for overlay (ex. 425/60; em. 480). Fluorescent cells were counted in ImageJ (Fiji) using the automated cell count function. The raw images were converted to an 8-bit standardized format (threshold 30) and inverted before a watershed was applied to separate any cells in direct contact. The ratio of green or red to blue cells was calculated. To evaluate the effects of antibiotic treatments on cell viability, cells were grown with Tet or Dox at a certain concentration as mentioned previously without plasmid DNA. After incubation, the live cells were counted using a TC20 automated cell counter (Bio-Rad).

### ELISA (enzyme-linked immunosorbent assay) experiment

The antibiotic-supplemented diets were fed to the flies at both larval and adult stages (L+/A+), or only at the larval stage (L+/A−). For the latter, the pupae were collected and rinsed with ddH_2_O twice before transferring to antibiotic-free vials. To test the maternal or paternal effects, the newly emerged males and females were crossed with their WT partners for 1–2 weeks. The F1 generation was reared on the antibiotic-free diet. Then, 100 eggs (0–6 h collection) and 10 adult females (1 day old) from each cross were collected as test samples for ELISA (Elabscience Biotechnology). In addition, 10 WT adult females (1 day old) from antibiotic-free and Tet-100-containing diets were collected as negative and positive controls, respectively. Three biological replicates were used for each cross and control. A measure of 400 µL of trichloroacetic acid solution (1%) and beads (Lysing Matrix D Bulk, Cat. 6540-434, MPbio, France) were added to the sample tube, and the tissues were homogenized using a Precellys 24 homogenizer (for 20 s at 6000 rpm). The sample tubes were centrifuged at 4,000 g for 10 min at room temperature. Then, the supernatant (27.5 µL) was mixed with reconstitution buffer (82.5 µL), and 50 µL of the mixture was used for analysis. The standard solution (1.0 ppm) was diluted according to the manufacturer’s instructions. A measure of 50 µL of diluted standard solution and sample mixture was added per well of the pre-coated 96-well microtiter plate in duplicate; 50 µL of the antibody working solution was added to each well, and the plate was covered with a lid, gently oscillated for 5 s, and incubated with shading light at 37°C for 30 min. After incubation, the microplate wells were washed six times with 250 µL/well of washing buffer, 100 μL/well of streptavidin–horseradish peroxidase (HRP conjugate) was added, and the plate was incubated at 37°C for 30 min in the dark. Then, the plate was washed again as described previously, and 50 μL of each substrate reagent A and B was added sequentially per well. After incubation at 37°C in the dark for 15 min, the reaction was stopped by adding 50 μL/well of stop solution. Optical density (OD) at 450 nm and 630 nm of each well was measured as reference wavelength (TECAN microplate reader). The concentrations (ppb) were calculated as the OD value measured at 630 nm subtracted from that measured at 450 nm. The absorbance percentage was calculated using the following formula: absorbance (%) = A/A0 × 100% (A: average absorbance of the standard solution or sample; A0: average absorbance of 0 ppb standard solution). The average absorbance value from duplicate wells was added to the standard curve. The concentration calculated from the standard curve was multiplied by 8 (the dilution factor for the pretreatment of tissue/egg samples according to the manual) for the final concentration of the samples.

### Biological experiments

To evaluate the effect of antibiotic treatments on fly survival and development time (from embryo to adult), the diet supplemented with the indicated antibiotics was fed to WT-USA flies, and adults were crossed as previously described. A total of 100 eggs were collected and dechorionated before transferring them to antibiotic-free vials. Dechorionation was conducted by submerging eggs into 50% bleach solution (Dan Klorix, Colgate-Palmolive, Hamburg, Germany) for 3.5 min. Then, the eggs were rinsed three times with distilled water to remove potential bleach residues. The number and the development time (days) of the resulting males and females were recorded. Eggs without dechorionation were tested in a similar manner as the control. To test the antibiotic treatments on the female lethality of FK strains, the diet with Tet-100 or Dox-100 was fed to the V229_M4f1 heterozygous larvae. The pupae were collected as described previously, and the newly emerged males and females (<4 h) were kept separately in the antibiotic-free vials for 1, 5, or 10 days before they were crossed with WT partners. This allowed different periods for antibiotic degradation in the flies. The flies were transferred daily to a fresh vial with a tetracycline-free diet for 9 days (10 vials in total). The fluorescent and non-fluorescent F1 adults (<24 h) from these 10 vials were sexed and counted. Meanwhile, the 1-day-old V229_M4f1 flies were crossed, and the offspring were reared and measured similarly, except that food with Tet-100 or Dox-100 was used throughout the experiments as controls. To assess the female survival, the V229_M4f1 larvae were fed with a diet containing Tet-100, Dox-100, or Dox-250, and the F0 adults were crossed on an antibiotic-free diet as described previously. The F1 transgenic females were collected every day in separated vials until no more females emerged. The females in those vials were counted every 2 days before transferring to a new vial (antibiotics-free) until all flies died. Meanwhile, the newly emerged F1 transgenic and WT males were counted to evaluate the male production under different antibiotic conditions. To verify the transgenerational effect of antibiotics, the diet with Tet-100 was fed to the V229_M8f2 heterozygous larvae, and the F0 and F1 transgenic females were crossed with WT males as described previously. The newly emerged F1 or F2 transgenic flies were sexed and separated into antibiotic-free food vials (20 flies per vial). These flies were counted every 2 days before transferring to new vials until 40 days or all flies died. The WT flies from antibiotic-free diet were counted similarly as the control. Here, three biological replicates were carried out for all the experiments.

## Statistics

Statistical analysis was carried out using SigmaPlot (v14.0). Differences in the green/blue or red/blue cell ratios, fly development or survival from different treatments, antibiotic concentrations (ELISA assays), or male production from different crosses were analyzed by one-way analysis of variance (ANOVA), and means (some are square root-transformed) were separated using either the Student–Newman–Keuls, Holm–Šidák, or Duncan’s method. Differences in the female lethality between the WT and transgenic offspring from different antibiotic-treated mothers or fathers were analyzed by the paired *t*-test. Differences in the survival rates of female offspring from antibiotic-treated mothers and fathers were analyzed using the z-test.

## Results and discussion

### Tet and Dox regulated the expression of Tet-off constructs *in vitro*

**FIGURE 1:**
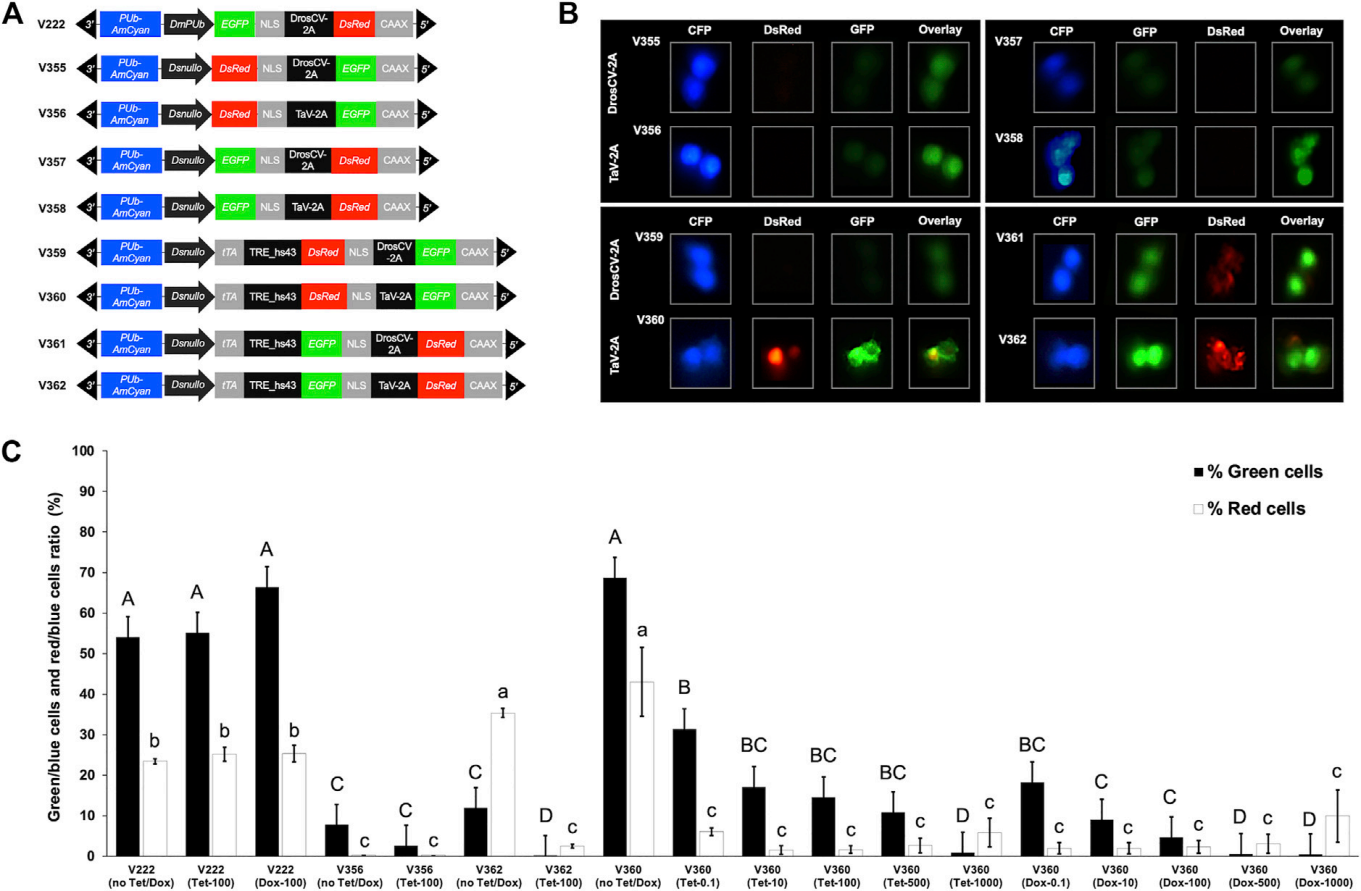
Effects of antibiotics on the expression of tetracycline-off (Tet-off) constructs in *Drosophila* S2 cells. **(A)** Schematic map of the *piggyBac* vectors containing a bicistronic gene cassette for EGFP-NLS (or DsRed-NLS) and DsRed-CAAX (or EGFP-CAAX), separated by a 2A peptide, expressed under the control of either the *D. melanogaster* PUb promoter (DmPUb), the *D. suzukii nullo* promoter (Dsnullo), or a TRE regulatory element that responds to Dsnullo-controlled tTA. Constructs contained DmPUb-AmCyan as an independent visible marker. **(B)** *Drosophila* S2 cells not treated with antibiotics and transfected with *piggyBac* vectors are shown. Images were taken under epifluorescent light conditions. **(C)** Ratios of green and red fluorescent cells relative to blue fluorescent cells are depicted, and the respective antibiotic treatments are indicated. Fluorescent cells were detected and counted using ImageJ, and the ratio of green/blue or red/blue cells was calculated. Each bar represents the mean ± SE of *n* = 3 experiments. Letters describing significant differences at *p* < 0.05 (one-way ANOVA or Student–Newman–Keuls method) are given for green (upper case) and red cell measurements (lower case).

We previously characterized promoters from four *Drosophila suzukii* cellularization genes, including *nullo*, *serendipity-α* (*sry-α*), *bottleneck* (*bnk*), and *slow-as-molasses* (*slam*). The *Dsnullo* promoter regulated the strongest *DsRed-NLS* gene expression in *Drosophila melanogaster* S2 cells (Yan et al., 2020a). We also previously generated *piggyBac* vectors containing a bicistronic gene cassette in which EGFP-NLS (or DsRed-NLS) and DsRed-CAAX (or EGFP-CAAX) were co-expressed by a 2A peptide (DrosCV-2A or TaV-2A) (Schwirz et al., 2020). The bicistronic gene cassette was regulated by the *Drosophila melanogaster polyubiquitin* (*DmPUb*) promoter, and *Drosophila suzukii* transformed by these *piggyBac* vectors showed whole-body green and red fluorescence (Schwirz et al., 2020). Here, we generated a range of *piggyBac* constructs in which the bicistronic gene cassette was either under the direct control of the *Dsnullo* promoter (V355–V358) or regulated by the TRE element that responds to *Dsnullo*-controlled tTA (V359–V362) (Figure 1A).

*Drosophila* S2 cells transfected with V355–V358 showed weak green fluorescence and no red fluorescence, regardless of the position of EGFP-NLS or DsRed-NLS to the 2A peptide (Figure 1B). Those cells also showed bright blue fluorescence driven by the *DmPUb* promoter (Figure 1B). The undetectable red fluorescence is possibly due to the relatively weak activity of the *Dsnullo* promoter compared to that of the *DmPUb* promoter and longer maturation time of the DsRed.T3 protein than EGFP (Bevis and Glick, 2002). Earlier *in vitro* studies showed that the combination of the tTA driver and TRE lethal effector components, but not a single component, mediated efficient cell death when antibiotics were absent (Schetelig and Handler, 2012a; Schetelig et al., 2021). Similarly, cells transfected with V360 or V362 (employing TaV-2A) showed intense green and red fluorescence (Figure 1B). This suggests a strong expression induction of the bicistronic gene cassette triggered by the binding of *Dsnullo*-regulated tTA to the TRE and efficient protein translation mediated by TaV-2A. On the other hand, cells transfected using V359 and V361 (employing DrosCV-2A) showed weak or moderate expression of EGFP-NLS and DsRed-NLS (Figure 1B). This suggested that the cleavage activity of DrosCV-2A is lower than that of TaV-2A when the bicistronic cassette is placed after TRE. Both DrosCV-2A and TaV-2A have been previously used to co-express two different pro-apoptotic genes including *reaper*, *head involution defective* (*hid*), and *grim*. S2 cell death assays showed that the lethality can be significantly enhanced by such binary expression compared to the single expression of a pro-apoptotic gene (Jaffri et al., 2020). Here, our observation suggested that TaV-2A may be preferred over DrosCV-2A for multiple expression of different lethal genes using the Tet-off system.

The weak expression of the reporter genes in V359 (DsRed-NLS_DrosCV-2A_ EGFP-CAAX) is possibly due to the position of DsRed relative to DrosCV-2A. It was found that translation was less efficient for DsRed when placed upstream of the 2A peptide than downstream (Wang et al., 2019). In addition, the cell imaging for V361 and V362 showed the loss of membrane integrity (Figure 1B), suggesting that DsRed aggregates disrupt cell walls. We previously identified a detrimental effect on cells *in vitro* and *in vivo* when overexpressing the DsRed.T3 with the membrane tag CAAX (Schwirz et al., 2020). Specifically, transgenic *Drosophila suzukii* expressing DsRed-CAAX showed extensive membrane blebbing and are homozygous lethal. Therefore, vectors containing DsRed-CAAX could be used to generate conditional lethal strains.

Since V360 and V362 exhibited strong expression of the reporter genes when no antibiotic was present, they were chosen to analyze the suppression of these genes in response to Tet or Dox treatments (Figure 1C). The V222 and V356, in which the bicistronic cassette is regulated by *DmPUb* and *Dsnullo* promoters, respectively, were also used as controls since they do not contain Tet-off components and thus should not respond to the antibiotic treatments. Since all tested constructs have a *DmPUb-AmCyan* cassette, the cells showing blue fluorescence suggested that these cells were transfected successfully. The number of red or green cells was then related to the number of blue cells to show the relative expression of DsRed or EGFP, which were controlled either by the *Dsnullo* promoter or TRE. When using V222 with no antibiotics, Tet-100 or Dox-100 for transfection, green and red cell ratios (related to blue cells) were in the range of 54.1%–66.4% and 23.4%–25.3%, respectively. The ratios for green or red cells under these treatments were not significantly different (*p* > 0.05, one-way ANOVA). Similarly, for V356, the green and red cells ratios were in the range of 2.6%–7.8% and 0.0%–0.1%, respectively, with no significant differences in these treatments (*p* > 0.05, one-way ANOVA). Therefore, the expression levels of the reporter genes in V222 and V356 were independent of the antibiotic treatments. On the other hand, the ratios of green and red cells for V362 were 11.9% and 35.3%, respectively, when the antibiotic was absent. Those ratios were significantly reduced to 0.1% and 2.5% (*p* < 0.05, one-way ANOVA), respectively, when Tet-100 was present. This indicated that for V362 the Tet at 100 μg/mL concentration effectively intercepted the binding between tTA and TRE, thereby suppressing the reporter gene expression. Since the DsRed-CAAX from V362 disrupted the integrity of the cell walls (Figure 1B), it is possible that cells were killed by such a detrimental effect and not counted in the assay.

Due to the detrimental effect of DsRed-CAAX on cells and high cleavage activity of TaV-2A, V360 (DsRed-NLS_TaV-2A_EGFP-CAAX) was used for further dose-dependent experiments. Without plasmid DNA, treatments with no antibiotics, Tet-0.1, Tet-10, Tet-100, Tet-500, Dox-0.1, Dox-1, and Dox-10 generated >70% alive cells, whereas treatments with Tet-1000, Dox-100, Dox-500, and Dox-1000 reduced the alive cells to 47%, 60%, 33%, and 27%, respectively (Supplementary File S2). This suggested that high doses of antibiotics reduce the cell viability. Alive cells transfected with V360 from different antibiotics treatments were further imaged and counted. The ratio of green/blue cells for V360 was 68.8% when no antibiotics were present (Figure 1C), similar to the corresponding ratio for V222. Adding Tet-0.1, Tet-10, Tet-100, or Tet-500 or Dox-0.1 significantly reduced the ratio of green/blue cells to 10.9%–31.4% (*p* < 0.05, one-way ANOVA). There was no significant difference among the green/blue cell ratios under these treatments (*p* > 0.05, one-way ANOVA). Adding Tet-1000, Dox-10, Dox-100, Dox-500, or Dox-1000 further reduced the ratio of green/blue cells to 0.5%–9.0%, significantly lower than that from the Tet 0.1 treatment (*p* < 0.05, one-way ANOVA). Meanwhile, the ratio of the red/blue cells was 43.0% when no antibiotic was present, significantly higher than that from V222 (*p* < 0.05, one-way ANOVA). Adding Tet or Dox at all tested concentrations significantly reduced the red/blue cell ratios to 1.5%–9.9% (*p* < 0.05, one-way ANOVA). There was no significant difference in the red/blue cell ratios under these treatments (*p* > 0.05, one-way ANOVA). Likely, the cell ratios from high-dose treatments such as Tet-1000, Dox-100, Dox-500, and Dox-1000 do not show the overall expression of reporter cassettes since fewer cells were viable compared to other treatments (Supplementary File S2). This may explain why these high-dose treatments did not further reduce the expression in a significant manner.

Since the translation efficiency may be hindered by placing DsRed before the 2A peptide and the DsRed protein needs a longer maturation time than EGFP (Bevis and Glick, 2002; Wang et al., 2019), the ratio of green/blue cells might be a better reference for the expression level of the bicistronic cassette than red cell counting. The results from green/blue cell ratios *in vitro* assays collectively suggested the following: 1) the expression of Tet-off constructs was sensitive to antibiotic treatments since low Tet or Dox levels (0.1 μg/mL) effectively reduced the fluorescent cells; 2) the high levels of Tet or Dox (500 or 1,000 μg/mL) were required to get the maximal suppression of reporter gene expression; 3) Dox exhibited overall higher efficiency than Tet in suppressing the reporter gene expression; 4) while the *Dsnullo* promoter itself mediated low reporter gene activity (indicated by the low ratio of green/blue cells from V356), the Tet-off system using the same promoter for tTA increased the reporter gene expression to a level similar to using a strong *DmPUb* promoter (ratio of green/blue cells from V222).

### Feeding Tet to *Drosophila suzukii* WT flies showed different effects on the development and survival of their offspring

**FIGURE 2:**
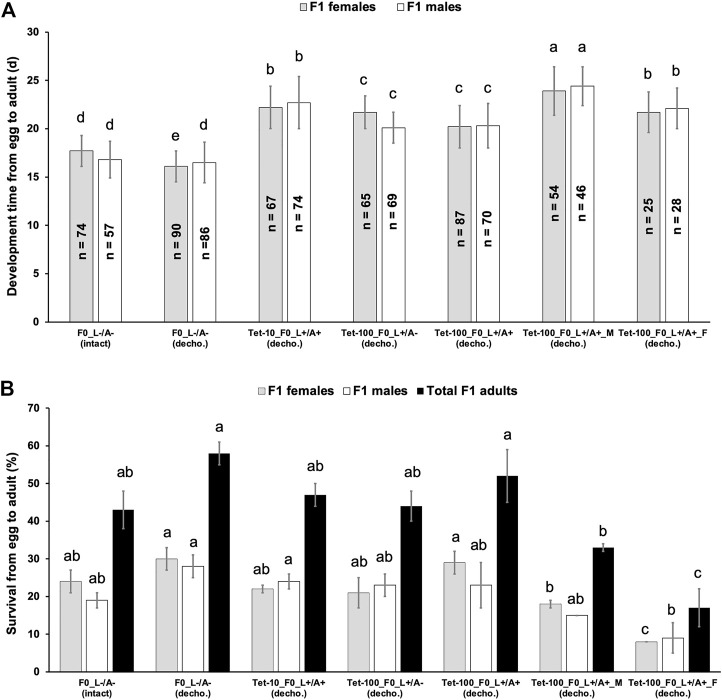
Effects of parental antibiotics on the development time **(A)** and survival **(B)** of the *D. suzukii* WT-USA strain. The flies from the parental generation (F0) were fed with food containing 10 or 100 μg/mL tetracycline (Tet-10 or Tet-100) at both larval and adult (L+/A+) stages or only at the larval stage (L+/A−) or were always fed with food containing no antibiotics (L−/A−). Then, 100 dechorionized (decho.) or intact eggs were transferred to an antibiotic-free vial, and the developmental time (days) from egg to adulthood as well as the number of newly emerged F1 flies was recorded. n represents the tested individuals. The mean and standard error from three replicates are shown. Different lower-case letters above the bars from the same kind of F0 flies indicate significant difference (*p* < 0.05, one-way ANOVA).

Tet at 100 μg/mL (Tet-100) was commonly used to rear the insect strains in which lethality is controlled by Tet-off systems (Gong et al., 2005; Schetelig et al., 2009; Ant et al., 2012; Schetelig and Handler, 2012a; Schetelig and Handler, 2012b; Jin et al., 2013; Yan and Scott, 2015), and Tet at 10 μg/mL (Tet-10) or lower was shown to be sufficient to maintain lethal strains of *Drosophila melanogaster* and *C. capitata* (Horn and Wimmer, 2003; Schetelig et al., 2009; Li et al., 2014; Upadhyay et al., 2022). In an SIT-like control program using transgenic sexing strains based on Tet-off systems, the last generation before release will be switched to non-Tet diet to produce male-only populations. For the sexing strains engineered with late lethality, Tet is often supplied to the adults, and rearing the offspring without Tet would trigger the designed lethality (Heinrich and Scott, 2000; Thomas et al., 2000; Fu et al., 2007). For the strains engineered with embryonic lethality, Tet should only be supplied to larvae but not adults to minimize the parental Tet that could suppress the early lethality in the offspring (Schetelig and Handler, 2012a; Schetelig and Handler, 2012b; Yan and Scott, 2015). Here, the impact of parental Tet on the fitness of the offspring using our WT-USA strain was tested. To avoid any Tet that might be passed on from the parental flies, the eggs collected from different treatments were dechorionized using a bleach-based method. By applying non-antibiotic conditions and dechorionation (L−/A−_decho), the development time (from egg to adult) of the female and male offspring was 16.1 and 16.5 days, respectively, which were comparable to those (17.7 and 16.8 days for female and male offspring, respectively) from non-antibiotic conditions, but without dechorionation (L−/A−_intact). Compared to the “L−/A−” treatment, feeding Tet-10 at larval and adult stages (Tet-10_L+/A+), feeding Tet-100 at only the larval stage (Tet-100_L+/A−), feeding Tet-100 at larval and adult stages (Tet-100_L+/A+), and feeding Tet-100 only to males (Tet-100_L+/A+_M) or females (Tet-100_L+/A+_F) delayed the developmental time of females to 22.2, 21.7, 20.2, 23.9, and 21.7 days, respectively (*p* < 0.001, one-way ANOVA; Figure 2A). Similarly, these treatments delayed the developmental time of male offspring to 22.7, 20.1, 20.3, 24.4, and 22.1 days, respectively (*p* < 0.001, one-way ANOVA). On the other hand, there was no significant difference in the survival of female or male offspring from treatments involving F0 sibling crossing, such as “L−/A−_intact,” “L−/A−_decho,” “Tet-10_L+/A+,” “Tet-100_L+/A−,” and “Tet-100_L+/A+,” despite the antibiotic treatments (*p* > 0.05, one-way ANOVA; Figure 2B). However, treatments involving inter-population crossing, including “Tet-100_L+/A+_M” and “Tet-100_L+/A+_F,” significantly reduced the number of their offspring (*p* < 0.05, one-way ANOVA; Figure 2B).

The development and survival (from egg to adult) of *Drosophila suzukii* is largely determined by variables for rearing such as temperature, humidity, and diet (Winkler et al., 2020). A summary from multiple studies showed that at temperatures close to 24 or 25°C, but different humidity and diet, the developmental times were in the range of 10–13 days and the survival rate were at 55%–85% (Winkler et al., 2020). Under our non-antibiotic conditions, the developmental time was at 16–17 days and thus longer compared to Winkler et al., possibly due to the humidity, diet and/or handling differences. Nevertheless, feeding Tet to the parents or only to the parental males or females delayed the development time of the offspring for 3.6–7.9 days (Figure 2A). This suggested some parental effects induced by the Tet treatment in the adults and/or an effect of the maternal Tet. To detect the presence of antibiotics in the treated flies and their offspring, ELISA was performed based on a Tet antibody. The assay detected 34.8 ng/g Tet in adult females that fed on Tet-100 at both larval and adult stages (Supplementary Figure S1). However, the amount of antibiotics in the eggs from different treatments, including different antibiotics (Tet or Dox), different concentrations (10, 100, or 250 μg/mL), different treated stages (larvae or adults), and treating males or females, were calculated and compared to non-antibiotic controls (Supplementary Figure S1; *p* > 0.05, one-way ANOVA). Such levels (0.5–3.4 ng/g) are also below or close to the detection limit (2.4 ng/g) of the ELISA kit. The results indicated that either antibiotics were not deposited into the eggs by their parents, or the ELISA kit is not suitable to detect antibiotics in *Drosophila* eggs.

While all treatments involving antibiotics delayed the developmental time in the next generation (Figure 2A), most of them did not affect the fly survival (Figure 2B). For a control program of *D. suzukii* using insect strains carrying Tet-off systems, these results would mean that the insect production in the pre-release generation should not be negatively affected by the Tet diet that was fed to the parental generation. Interestingly, crossing Tet-treated flies with their untreated partners resulted in a severe reduction in the offspring number (Figure 2B). Since feeding Tet to both parents should have stronger effects (if not the same) on the performance of their offspring compared to only feeding Tet to one parent, it is unlikely that the Tet treatment to father or mother caused the reduction in the offspring number. *Drosophila* species are known for assortative mating, which describes the phenomena that individuals mate assortatively with others of similar environmentally induced phenotypes (Robinson et al., 2012; Najarro et al., 2015). We also observed strong assortative mating among different *D. suzukii* populations in our laboratory (data not shown). Such assortative mating may promote the sexual reluctance between individuals from antibiotic-treated and -untreated populations. It is possible that some eggs collected from such crosses were not fertilized (even if the flies were crossed for >1 week) and therefore never hatch. On the other hand, for all treatments which showed no impact on the offspring survival, siblings from the same population were used for the crosses, and thus females were more likely to mate. The assortative mating was reported as an obstacle for the SIT application against the melon fly (Hibino and Iwahashi, 1991; Koyama et al., 2004). Therefore, the assortative mating between the mass-rearing population using antibiotic-supplemented diets and the targeted wild *D. suzukii* populations with no or limited contact to antibiotics should be investigated for the implementation of any genetic control strategies that are based on antibiotic-fed systems. One useful tool for such purpose is the sperm-marking strain that can be employed to monitor the (assortative) mating among certain populations (Ahmed et al., 2019; Yan et al., 2021).

### Feeding Dox to F0 flies inhibits the F1 female lethality in the *D. suzukii* FK strain V229_M4f1

**FIGURE 3:**
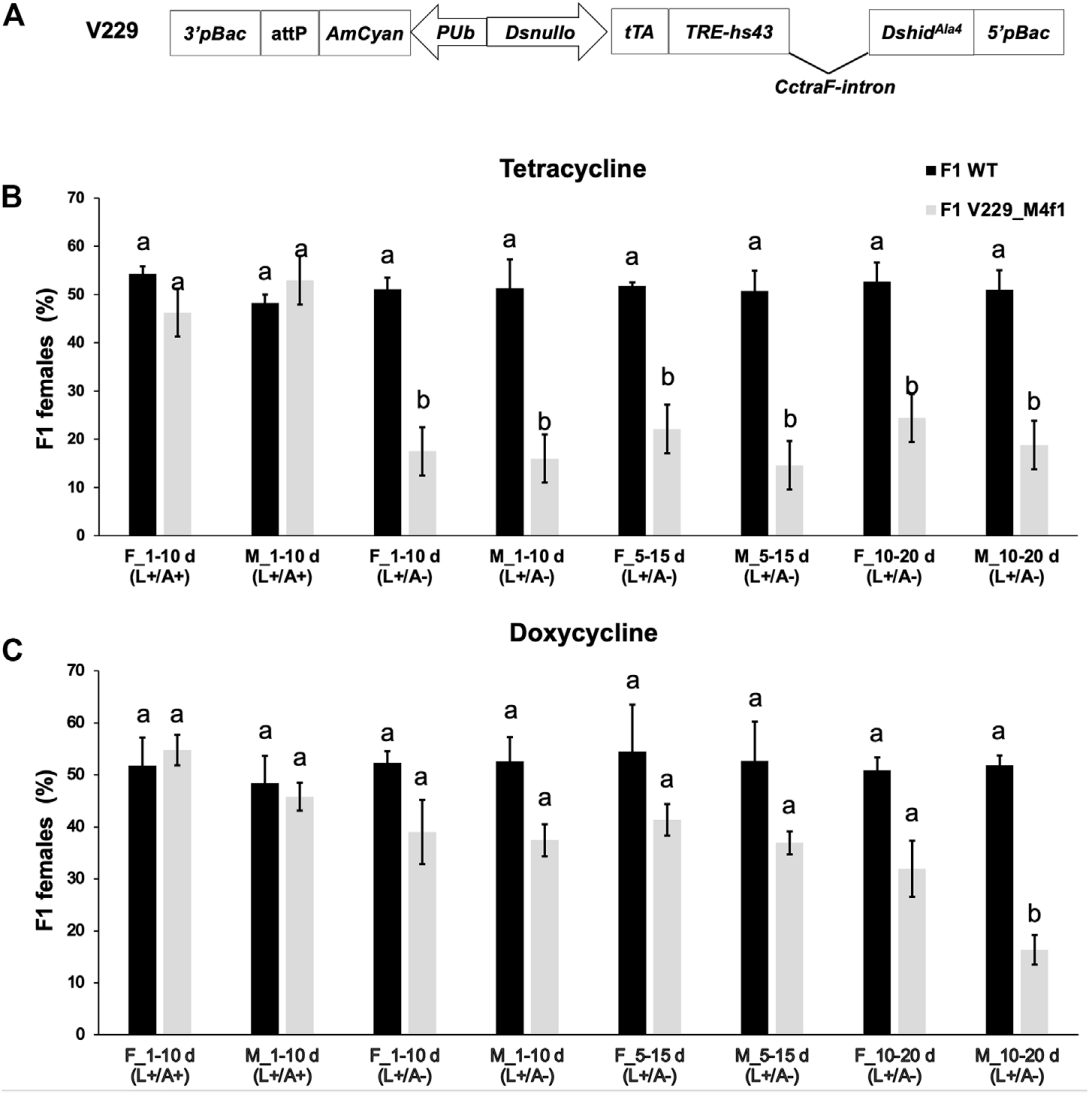
Parental effect of antibiotics on the female lethality of the *Drosophila suzukii* female-killing strain V229_M4f1. **(A)** V229_M4f1 was previously generated by the germline transformation using the vector V229*_pBXLII_attP_PUbAmCyan_Dsnullo-tTA-SV40_TREhs43-Dshid* ^*Ala4*^ *-CctraF-SV40* which contains the female-specific *transformer* intron from *C. capitata* (*CctraF*), as well as an AmCyan marker gene regulated by the constitutive *D. melanogaster polyubiquitin* (*PUb*) promoter and an *att*P recombination site (Schetelig et al., 2021). Transgenic larvae (heterozygous) from the parental generation (F0) were fed on diets containing 100 μg/mL tetracycline **(B)** or doxycycline **(C)**. The newly emerged males (M) and females (F) were separated into different antibiotic-free vials, and 1-, 5-, or 10-day (d)-old males and females were crossed with their WT partners (4–7 days old) on antibiotic-free diet. Crosses were maintained for 10 days by transferring the flies to a fresh vial (antibiotic-free diet) every day. The fluorescent and non-fluorescent (WT) F1 adults from these ten vials were sexed and counted. Similarly, control experiments were carried out using 1-day-old transgenic flies and antibiotic-treated diets. +/−, antibiotic-treated diet (100 μg/mL) used for F0 larvae before switching to an antibiotic-free diet for F0 and F1 flies; +/+, antibiotic-treated diet (100 μg/mL) used for all F0 and F1 stages. The mean and standard errors from three replicates are shown. Letters above the bars indicate significant differences (*p* < 0.05, paired *t*-test).

In the *in vitro* study using Tet-off constructs, applying high levels of Tet or Dox (500 or 1, 000 μg/mL) could minimize the effector gene expression (Figure 1C). However, such treatments had severe negative impact on the cell viability (Supplementary File S2) as well as the fitness of *D. suzukii* flies *in vivo* (Schetelig et al., 2021). Therefore, they may not be considered for practical use. Earlier studies suggested that the amount of Tet needed to suppress the Tet-off system-mediated lethality was subjected to insect species and strains with certain transgene activity. While Tet at 100 μg/mL was commonly used as previously mentioned, in other species and Tet-off systems, higher concentrations up to 200 μg/mL were needed to completely suppress the lethality (Tan et al., 2013; Concha et al., 2020). At even higher concentrations (300 μg/mL), the insect fitness of *C. capitata* and *Anastrepha suspensa* was severely affected (Schetelig et al., 2009; Schetelig and Handler, 2012a). Therefore, we selected 100 and 250 μg/mL as test concentrations for most experiments. The *Drosophila suzukii* FK strain V229_M4f1 was previously generated by germline transformation using the vector V229*_pBXLII_attP_PUbAmCyan_Dsnullo-tTA-SV40_TREhs43-Dshid* ^*Ala4*^ *-CctraF-SV40* (Schetelig et al., 2021). The V229_M4f1 strain showed moderate lethality and killed females mostly at the pupal and adult stages when Tet was not present. Here, we fed Tet-100 or Dox-100 to V229_M4f1 larvae, separated the newly emerged males and females, and crossed them at different ages with their WT partners on non-antibiotic food. Previously, antibiotic degradation was observed in the RIDL strain (OX513A) of the yellow fever mosquito *Aedes aegypti* (Curtis et al., 2015), possibly due to the insect metabolism or the binding of large amounts of Tet by high concentrations of the tTA protein produced in RIDL systems. Therefore, flies crossed at an older age were expected to pass lower amounts of antibiotics to their offspring due to degradation. Since heterozygous V229_M4f1 flies were crossed, half of the progeny were WT with a 1:1 sex ratio (Figure 3), while the other half were transgenic flies (heterozygous) with a sex ratio subject to the parental antibiotics. Previously, crossing V229_M4f1 males with WT females on non-Tet diet produced transgenic offspring consisting of only 14.2% females (Schetelig et al., 2021). When Tet-100 was used here, regardless of the F0 sex and age, the female percentages of transgenic adults were in the range of 14.6%–24.5%, which were significantly lower than those of WT in each cross (*p* < 0.05, paired *t*-test) (Figure 3A). Therefore, feeding Tet-100 to F0 flies did not rescue the female lethality in F1. When using Dox-100, most of the female percentages of transgenic flies were in the range of 31.9%–41.3%, not significantly different from those of WT flies (*p* > 0.05, paired *t*-test) (Figure 3B). Such observation suggested that the F1 female lethality was suppressed, possibly due to some Dox-induced parental effects and/or an effect of the inherited Dox. The only exception was the cross using 10–20-day-old V229_M4f1 males, which again produced transgenic offspring with a strong male-biased sex ratio (the female percentage was 16.4%), indicating that either Dox degraded faster in males or males contributed less amount of Dox to the offspring than females at this age. When both F0 and F1 generations were maintained on Tet or Dox diet, no female lethality was observed in the transgenic F1 flies.

For *D. melanogaster*, feeding Tet (10–1,000 μg/mL) to adults suppressed the Tet-off system-mediated embryonic lethality, while Dox was more efficient at identical concentrations (Horn and Wimmer, 2003). In *C. capitata*, feeding Dox instead of Tet at 100 μg/mL to adult flies completely suppressed embryonic lethality (Schetelig et al., 2009). Meanwhile, for the fsRIDL strain of the malaria mosquito *Anopheles stephensi*, feeding less than 10 μg/mL of Tet to the larvae was not sufficient to completely rescue the flightless phenotype of the females (Marinotti et al., 2013). For the *Aedes aegypti* OX513A strain, feeding 1 μg/mL Dox to the larvae fully rescued the pupal lethality and flightless phenotype, which was about 100 times more effective than Tet (Curtis et al., 2015). Here, we showed that feeding Dox, but not Tet, to *D. suzukii* F0 larvae led to suppression of female lethality in F1 (Figure 3). Together these results suggested that Dox is a more efficient suppressor molecule than Tet for drosophilid, tephritid, and mosquito species. Interestingly, for *Aedes aegypti* OX513A, adult male or female receiving 50 or 100 μg/mL chlortetracycline only produced very few adult progeny (Curtis et al., 2015), suggesting that either the antibiotics were not passed to the offspring or they were inherited but not able to suppress the engineered lethality. Since the lethality of OX513A strain is based on the accumulation of tTA which kills mosquitoes at the larval stage (Phuc et al., 2007; Curtis et al., 2015), it is possible that the chlortetracycline was inherited but degraded and therefore not able to bind and deactivate tTA binding at a later stage. On the other hand, in the early-lethal systems in *D. melanogaster* and *C. capitata* based on embryonic-specific promoters, parental antibiotics were suspected as a possible cause for the suppression of the embryonic lethality (Horn and Wimmer, 2003; Schetelig et al., 2009).

### Feeding Tet or Dox to F0 mothers generated long-lived F1 female survivors in the FK strain V229_M4f1

In the previous study, several *Drosophila suzukii* female-killing lines produced few female adults when parental males were fed with antibiotics at larval stages, but survivors quickly died (Schetelig et al., 2021). For example, the female lethality in the strain V229_M41f1 reached 100% at day 1 after the emergence (1 day), whereas for strain V229_M37f2 and V229_M39m1, it reached 100% at day 3. However, for the strain V229_M4f1, the female lethality was only investigated on day 1 but not beyond that stage in the earlier study. Here, we counted the survival rates of the V229_M4f1 females that derived from antibiotic-treated (at the larval stage) mothers or fathers. When Tet-100 was added to the diet, 5% and 0.4% of females related to the treated fathers survived until days 2 and 4 after emergence, respectively, which were significantly lower than those from treated mothers (*p* < 0.05, z-test) (Table 1). All females derived from Tet-treated fathers died until day 6, while three females from Tet-treated mothers survived until day 6 and one female until day 28. When Dox-100 was applied, 3% of the females descending from treated fathers survived until day 2 and all died on day 4, while significantly higher survival rates at these days (21.4% and 5.8%) were found in the crosses using the treated mothers (*p* < 0.05, z-test). When Dox-250 was applied, no females from the treated fathers survived until day 2, whereas 18 females from the treated mother survived to this day and one female until day 43.

**TABLE 1:** Effects of parental antibiotics on the female survival rates in the *D. suzukii* FK strain V229_M4f1.

Antibiotic^a^ concentration	F0 flies^b^	F1 female survival (%)^c^				
		Day 2	Day 4	Day 6	Day 8	Day 10
Tet-100	Female	13.8 a (22/160)	3.7 a (6/160)	1.9 a (3/160)	1.3 (2/160)	0.6 (1/160)^d^
	Male	5.0 b (11/218)	0.4 b (1/218)	0 a (0/218)	—	—
Dox-100	Female	21.4 a (22/103)	5.8 a (6/103)	3.9 (4/103)	1.0 (1/103)	0 (0/103)
	Male	2.1 b (3/143)	0 b (0/143)	—	—	—
Dox-250	Female	25.8 a (18/70)	4.3 (3/70)	1.4 (1/70)	1.4 (1/70)	1.4 (1/70)^e^
	Male	0 b (0/94)	—	—	—	—

^a^ The diet containing the indicated antibiotics was fed to the transgenic F0 larvae (heterozygous), and the antibiotic-free diet was used afterward.

^b^ The F0 flies (1 day old) were crossed with their wild-type (WT) partners (4–7 days old) on an antibiotic-free diet for successive 10 days.

^c^ The number of F1 female survivors on certain days relative to the total number of the newly emerged F1 females from three replicates. Different lower-case letters between the F1 survival rates from F0 females and males with the same antibiotic treatment indicate significant differences (*p* < 0.05, z-test).

^d^ This female died on day 28.

^e^ This female died on day 43.

It appears that line V229_M4f1 kills females through the development, and a complete penetrance of the lethal phenotype can be achieved at a young adult stage when only feeding antibiotics to the father. On the other hand, feeding antibiotics to the mother may be responsible for the “escaper” of the lethal system that survived to the late adult stage. Treated mothers may load more antibiotic molecules into some eggs than others, so they become more resistant to the lethal system. Therefore, strategies like fsRIDL or TESS, which generated male-only populations, might be more advantageous than bi-sex lethal systems, which do not eliminate females in the release generation. Several release programs for mosquito control combined the bi-sex lethal RIDL strains with the sexing procedure that mechanically removes most females (Harris et al., 2011; Carvalho et al., 2014). However, for the insect species without an efficient sexing method, bi-sex lethal systems need to be carefully evaluated for the effect of maternal antibiotics on the penetrance of the killing effect.

### Feeding Tet or Dox to F0 flies showed different effects on the F1 male production in the strain V229_M4f1

Similarly, we also counted the males derived from antibiotic-treated V229_M4f1 heterozygous mothers or fathers. Among all treated crosses, the Tet-100 fathers produced the highest number (262 ± 51) of male adult offspring, which was similar to that of WT (*p* = 0.410, one-way ANOVA). In contrast, the Dox-250 mothers produced the lowest number of males (68 ± 19), which was significantly lower than those from all other crosses (*p* < 0.05, one-way ANOVA; Table 2). In addition, all treated mothers and Dox-treated fathers produced fewer males compared to WT crosses (*p* < 0.05, One-way ANOVA), suggesting that the antibiotic residues from those treatments harmed the male survival throughout development. More specifically, the number of transgenic males descending from Dox-250-fed heterozygous mothers and fathers was significantly lower than that from Tet-100-fed mothers and fathers, respectively (*p* < 0.05, one-way ANOVA). Meanwhile, the number of WT male siblings in Dox-250 and Tet-100 were not significantly different (*p* > 0.05, one-way ANOVA). These observations indicated that the male survival in the transgenic files was more sensitive to increased doses of Dox than that in the WT flies, possibly due to fitness costs of the transgene. Male production is an important parameter to evaluate the suitability of insect strains for an SIT program. The results suggested that feeding Dox to the *D. suzukii* FK strains at higher concentrations may reduce male production.

**TABLE 2:** Effects of antibiotics on the male production of the *Drosophila suzukii* FK strain V229_M4f1.

Antibiotic^a^ (concentration)	F0 flies^b^	No. of F1 males^c^		
		FL	Non-FL	Total
Tet +/−(100 μg/mL)	Female	96 ± 18 ab	106 ± 18 ab	202 ± 35 b
	Male	126 ± 23 a	136 ± 31 a	262 ± 51 ab
Dox +/−(100 μg/mL)	Female	79 ± 4 ab	101 ± 16 ab	180 ± 16 b
	Male	83 ± 10 ab	97 ± 33 ab	180 ± 43 b
Dox +/−(250 μg/mL)	Female	24 ± 1 c	44 ± 18 b	68 ± 19 d
	Male	63 ± 16 bc	81 ± 6 ab	144 ± 21 bc
WT (no antibiotics)	—	—	—	297 ± 13 a

^a^ The diet containing the indicated antibiotics was fed to the transgenic F0 larvae (heterozygous), and the antibiotic-free diet was used afterward.

^b^ The F0 flies (1 day old) were crossed with their wildtype (WT) partners (4–7 days old).

^c^ The number of the newly emerged F1 males on certain days (after eclosion) was scored. Different lower-case letters in the same column indicate a significant difference (*p* < 0.05, one-way ANOVA).

### Feeding Tet to F0 mothers delayed the lethal effect for one generation in the FK strain V229_M8f2

It was recognized that the parental antibiotics might suppress the lethality correlated with the pro-apoptotic gene’s activity or expression level (Horn and Wimmer, 2003; Schetelig et al., 2009; Yan and Scott, 2015; Yan et al., 2017). The latter is further subjected to the positional effects of the transgene (Schetelig et al., 2016; Concha et al., 2020; Yan et al., 2020c). It is possible that the low female lethality that we previously observed in some V229 lines was due to the low or moderate activity of the pro-apoptotic gene that was further suppressed by parental Tet (Schetelig et al., 2021). Therefore, we selected line V229_M8f2 that showed the lowest female lethality (32.7%) among seven V229 lines in the earlier study for a transgenerational longevity test. Similarly, Tet-100 was fed to F0 larvae, and F0 or F1 females were crossed with WT males on non-antibiotic food. We found that the survival rates of the F2 males on days 11, 21, and 31 were 65.0%, 53.3%, and 45.0%, respectively, which were not significantly different from those of WT males (*p* > 0.05, one-way ANOVA). Meanwhile, the survival rates of the F1 females on days 11, 21, and 31 were 88.3%, 68.3%, and 26.6%, respectively, which were not significantly different from those from WT females (*p* > 0.05, one-way ANOVA). However, most of the F2 females died on day 3, indicated by the 26.7% survival rate, which was significantly lower than those from the F1 females (91.7%) or WT females (95%) (*p* < 0.05, one-way ANOVA). This resembled the lethal adult stage in the F1 generation that we observed in most V229 lines (Schetelig et al., 2021). Additionally, all F2 females died on day 19, indicating that the full penetrance of the lethality can be achieved for this line after one additional generation after the last antibiotic treatment.

A previous study also reported that for *D. melanogaster* strains carrying a Tet-off-mediated fsRIDL system, feeding parents with 10 μg/mL Tet was as efficient as 100 μg/mL Tet in suppressing the female lethality in F1. However, the latter delayed the female lethality for one generation (Upadhyay et al., 2022). Homozygous strains were stably kept for recently developed transgenic sexing strains in *D. suzukii* using the pro-apoptotic gene *Dmhid* ^*Ala5*^ or *Dsrpr* as lethal effectors on diet with 20 μg/mL Tet (Li et al., 2021). Therefore, a low dose of Tet rather than a high dose may be preferred for *Drosophila* species in such laboratory experiments. This would provide a better assessment toward the induced lethality from different strains by minimizing the effects of the antibiotics.

## Conclusion

For genetic pest control strategies such as RIDL or TESS, a proper Tet-feeding scheme that responsively and efficiently controls the lethal system is critical for applications in the mass-rearing and field release programs. Since those lethal systems could be leaky due to the insufficient or degraded Tet that does not entirely suppress the expression of the lethal gene (Thomas et al., 2000; Horn and Wimmer, 2003; Schetelig et al., 2009; Marinotti et al., 2013; Tan et al., 2013; Curtis et al., 2015; Yan and Scott, 2015; Schetelig et al., 2016; Concha et al., 2020), it is attractive to use higher doses of antibiotics for the maintenance of stock populations to counteract such potential leakiness of lethality. Here, we showed that while a high dose of antibiotics reduced the expression of Tet-off constructs to minimal in cell culture experiments, such treatments may have a profound effect on the performances of the *D. suzukii* transgenic strains employing the Tet-off system. We verified that feeding Dox but not Tet to F0 flies suppressed the F1 female lethality in the FK strain V229_M4f1. We also found that the parents treated with Dox produced significantly less transgenic male offspring than those from other antibiotic treatments. A genome-wide study showed that among several Tet derivatives, Dox elicited the most significant responses in the gene expression in *Saccharomyces cerevisiae* (Sanchez et al., 2020), and it also substantially alters cellular metabolism and impairs mitochondrial function (Luger et al., 2018). Therefore, although Dox is a more efficient suppressor molecule than Tet, it might undermine the lethal system at the designated stage and have a more severe impact on strain fitness.

**FIGURE 4:**
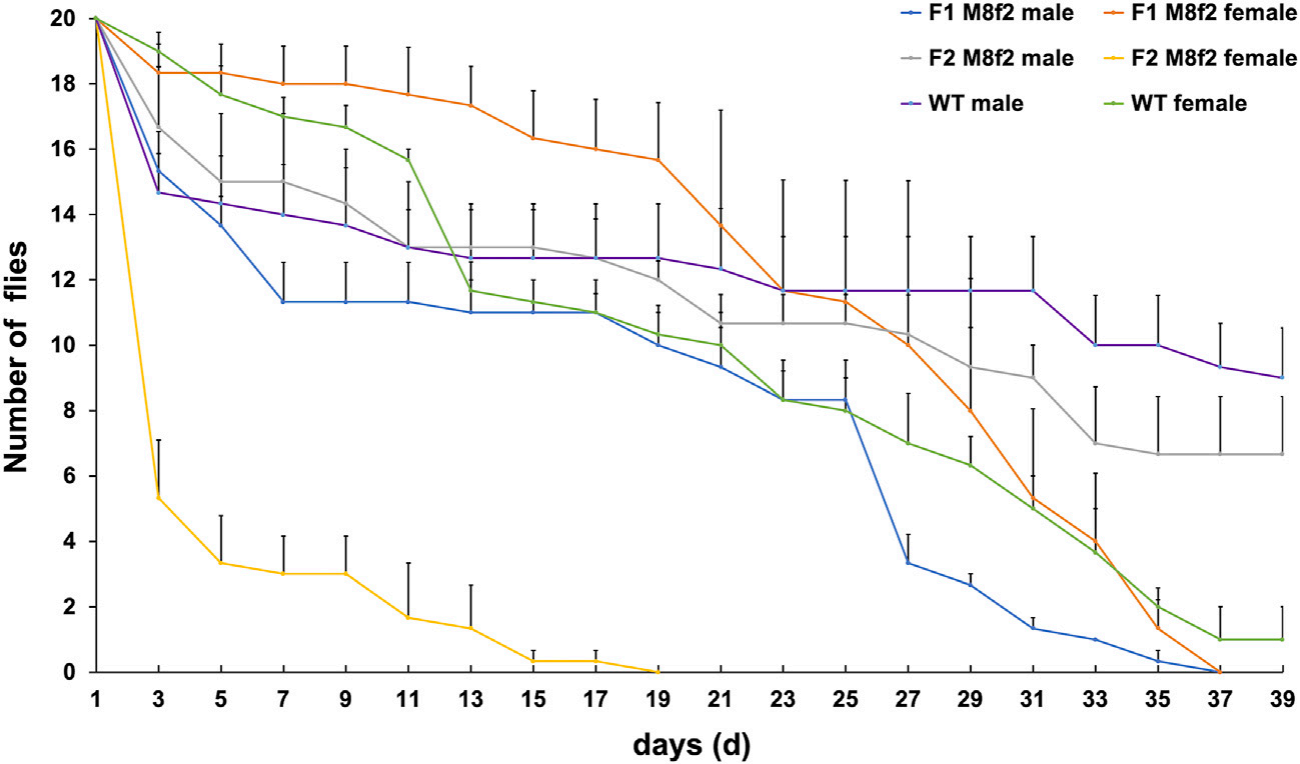
Transgenerational effect of antibiotics on fly longevity in the *D. suzukii* FK strain V229_M8f2. V229_M8f2 was previously generated by the germline transformation using the vector V229*_pBXLII_attP_PUbAmCyan_Dsnullo-tTA-SV40_TREhs43-Dshid* ^*Ala4*^ *-CctraF-SV40* (Schetelig et al., 2021). Tet-100 diet was fed to V229_M8f2 F0 larvae but not to later stages. F0 or F1 females were crossed with the WT males, and newly emerged transgenic F1 and F2 flies were sexed and separated into fresh diet vials (20 flies per vial). These males or females were counted every 2 days before transferring to new vials until day 40 or all flies died. WT flies from the non-Tet diet were counted similarly. The mean and standard errors from three replicate experiments are shown.

The Tet-off constructs for *in vitro* analysis contain the same driver cassette (*Dsnullo-tTA*) and TRE-hs43 as in the V229 construct that was used to generate the FK strains V229_M4f1 and V229_M8f2. The only difference is the effector part. While the 2A peptide reporter cassettes serve as visual markers for the *in vitro* experiments, the pro-apoptotic effector gene *Dshid* ^*Ala4*^ in V229 allows us to evaluate the transgene expression by scoring the female lethal phenotype. Collectively, we showed that (more) female survivors could rise in our FK strains under certain antibiotic treatments. For the strain V229_M4f1 which showed moderate transgene activity, feeding Dox to F0 males or females suppressed the female lethality in F1, and feeding Tet or Dox to F0 females (but not males) generated long-lived F1 females. For the strain V229_M8f2 which showed weak transgene activity, feeding Tet to F0 females delayed the female lethality to F2. The suppression of F1 lethality observed in our FK strains was likely due to the effects of antibiotics that inherited in this generation, as previously suggested in several studies (Horn and Wimmer, 2003; Schetelig et al., 2009; Ogaugwu et al., 2013; Yan and Scott, 2015; Upadhyay et al., 2022). Another plausible reason for the suppressed lethality that is linked to the parental sex is the parental effects such as parental imprinting. Parental imprinting refers to the phenomena that the expression of an allele differs based on the sex of the parent that transmitted the allele (Lloyd, 2000; Lemos et al., 2014). In *D. melanogaster*, imprinting is typically associated with heterochromatin or regions with specific chromatin structures and often leads to epigenetic silencing (Abbott et al., 2013; Lemos et al., 2014; McEachern et al., 2014). It is possible that the transgene in the tested V229 strains was inserted into a genomic region with an imprinting-prone chromatin structure; therefore, the male parent transmitted an active form of the transgene, whereas the female transmitted an inactive silenced form which led to F1 survivors (Table 1). In addition, transgenerational imprinting was also observed in *D. melanogaster* (Oh et al., 2020), which might be associated with the one-generation delayed lethality in the strain V229_M8f2 (Figure 4). Further studies can be conducted to investigate the effects of the parental imprinting on the transgene-based lethal systems, such as verification of the genomic site of the transgene and its chromatin structure, comparison for the epigenetic silencing of the transgene from parental male and female, and transgenerational effects of such imprinting on the transgene activities.

The potential resistances to the Tet-off-based lethal system likely depend on the mechanism of lethality as well as transgene activity from each certain strain. For practical application, strains with strong engineered lethality (but otherwise fit) might be preferred over weak ones for a high or complete penetrance of lethality in the mass-rearing scenario. The strong or redundant lethality could help to counteract the pre-existing resistance in the wild populations (Knudsen et al., 2020). However, it may also speed up the generation of the second-site resistance mutations which caused the genetic breakdown of RIDL lethality in *D. melanogaster* (Zhao et al., 2020). Therefore, incorporating two or more lethal effectors/systems into the insect strains intended for use may help ensure the complete penetrance of lethality and reduce the development of resistance (Jaffri et al., 2020; Yan and Scott, 2020; Li et al., 2021; Upadhyay et al., 2022). For any genetic control strategies employing the Tet-off system, excessive use of Tet or its derivatives could reduce the strain fitness or lead to survivors, which may cause undesired damage or reduce the control efficiency. Therefore, the overall effects of antibiotics, including parental and transgenerational effects, on the engineered lethality and insect fitness need to be carefully evaluated in a species- and strain-specific manner for a safe and efficient practical application. Together with other genetic control programs such as SIT and incompatible insect technique (Nikolouli et al., 2018; Sassù et al., 2020), genetically engineered lethal strains could facilitate the sustainable management of this global pest *D. suzukii*.

## Data Availability

The original contributions presented in the study are included in the article/Supplementary Material; further inquiries can be directed to the corresponding author.

## Supplementary material

SUPPLEMENTARY TABLE S1 Antibiotics effects on live cells.

SUPPLEMENTARY FIGURE S2 ELISA analysis to measure antibiotics in *D. suzukii* adults and eggs.
