# Supplementary figures and images for: Effects of antibiotics on the in vitro expression of tetracycline-off constructs and the performance of Drosophila suzukii female-killing strains
Source: Front Bioeng Biotechnol. 2023 Feb 14;11:876492. doi: 10.3389/fbioe.2023.876492 (PMC9971817; doi:10.3389/fbioe.2023.876492)

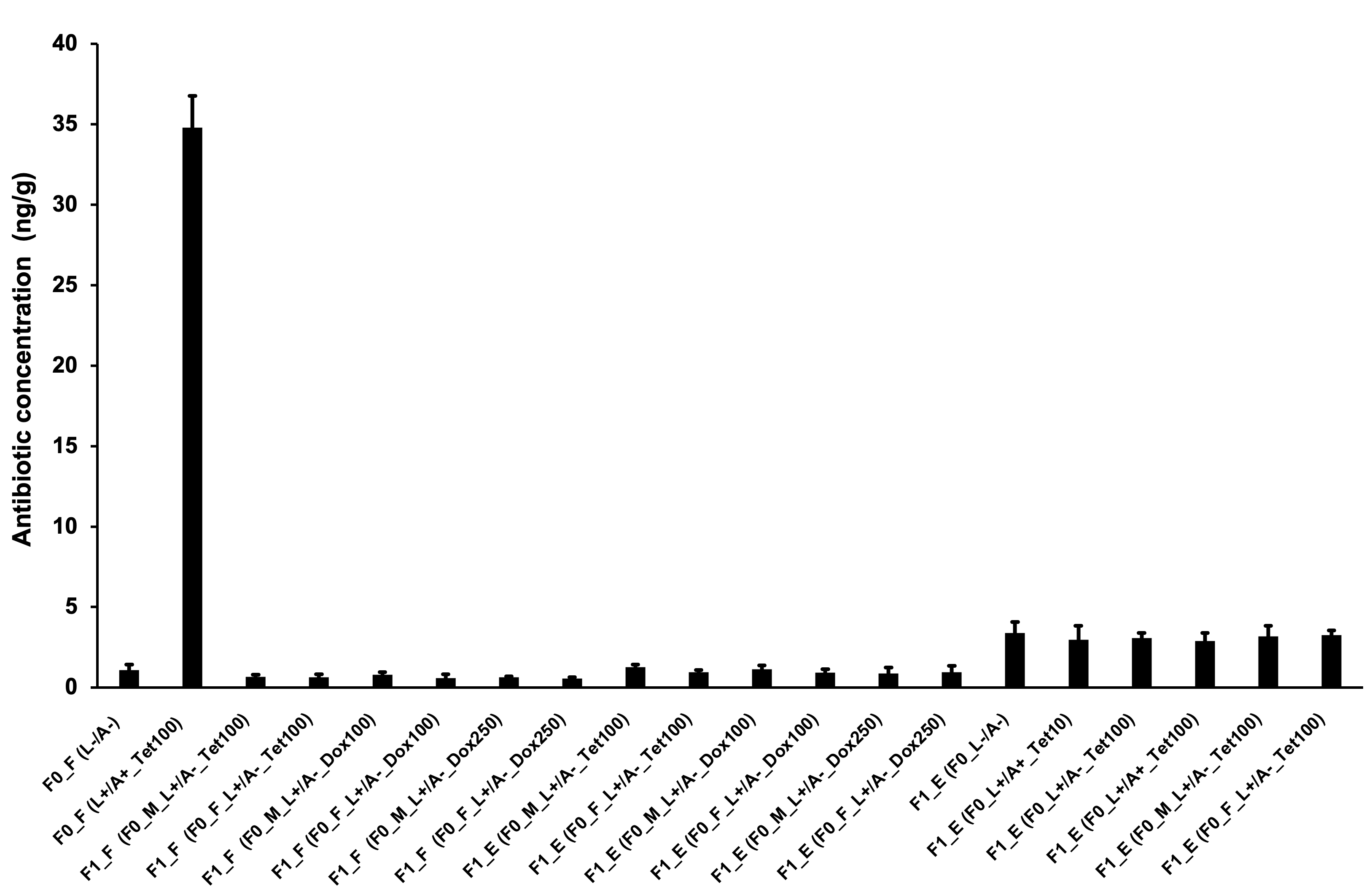

Supplement: Supplementary file 1 [file Image1.JPEG]
